# The KRAS, ATR and CHEK1 expression levels in endometrial cancer are the risk factors predicting recurrence

**DOI:** 10.1371/journal.pone.0302075

**Published:** 2024-04-26

**Authors:** Liubov Buchynska, Inna Gordiienko, Nadiia Glushchenko, Nataliia Iurchenko

**Affiliations:** Department of Cancer Genetic and Oncohematology, R.E. Kavetsky Institute of Experimental Pathology, Oncology and Radiobiology National Academy of Sciences of Ukraine, Kyiv, Ukraine; Tongji Medical University: Tongji Medical College, CHINA

## Abstract

Endometrial cancer is the most prevalent gynecologic malignancy with a high risk of recurrence. Local recurrence occurs in 7–20% of patients with treated stage I cancer within 3 years after primary treatment. In this study, we found significantly elevated mRNA expression levels of the oncoprotein *KRAS*, along with two replicative stress markers, *ATR* and *CHEK1*, in samples of endometrial carcinomas of endometrium (ECE) from patients with relapse. In contrast, mRNA expression levels of the studied genes were low and uniform in samples from patients without relapse. Elevated levels of KRAS protein and the phosphorylated form of ATR/CHEK1 were distinguishing features of recurrent ECE. A strong positive correlation was found between elevated mRNA and protein levels of the studied molecules. Elevated KRAS protein levels are characteristic of poorly differentiated (G3) endometrial carcinomas with deep myometrial invasion in patients without recurrence. In contrast, in patients with recurrence, higher protein levels of KRAS, pATR and pCHEK1 were observed in samples of G1-2 endometrial carcinomas, with statistically significant differences confirmed for pATR. High pCHEK1 protein levels are associated with deep tumor invasion in the myometrium among patients with recurrence. ROC analysis confirmed that evaluating the specificity and sensitivity of KRAS, pATR and pCHEK1 predicts recurrence development in patients with ECE. Our findings indicate that markers of replicative stress may play a significant role in ECE pathogenesis. Determining their levels in tumor samples after primary treatment could help define patients at high risk of recurrence and guide consequent courses of treatment.

## Introduction

Endometrial cancer (EC) is one of the most common malignancies in the field of female oncology, both in Ukraine and in other European countries [1, 2]. The high incidence of this cancer, especially among women of reproductive age, highlights the need for new methodologies to predict the course of the disease as a basis for preventing metastasis and choosing optimal treatment strategies. Approximately 80% of diagnosed cases of EC by histological type are endometrioid carcinoma of the endometrium (ECE), known for its favorable disease course. However, 7–20% of patients with stage I EC tumors experience metastases in regional lymph nodes within 3 years after primary treatment [1, 2].

Researchers have confirmed that categorizing patients into prognostic risk groups is crucial for selecting appropriate adjuvant therapy [2]. Stage I ECE, G1-2, with invasion in the myometrium less than 50% and intact lymphovascular space (LVS) corresponds to a low risk of progression, while stage I ECE, G1-2, with invasion in the myometrium of more than 50% and intact LVS is associated with an intermediate risk of progression. Stage I ECE, G3, with invasion in the myometrium higher than 50%, regardless of LVS involvement, is strongly associated with cancer progression. The molecular heterogeneity of EC underlies the clinical course variability and significantly impacts tumor progression and therapy effectiveness [3, 4].

Genomic and transcriptomic analyses conducted as part of The Cancer Genome Atlas Research (TCGA) project identified four molecular subtypes of ECE ("POLE—ultramutated"; MSI-hypermutated" with a high frequency of mutations in the KRAS and PTEN genes; "Copy number low" and "Copy number high"), each characterized by different malignancies and disease courses [2]. The ESGO/ESTRO/ESP group of experts has proposed new recommendations for the diagnostic and treatment strategies of patients with ECE. However, access to molecular testing is not uniform, and subsequent management (surgical, adjuvant therapy) is unacceptably variable. The authors emphasize that the specified classification does not fully reflect the heterogeneity of molecular changes that occur during EC progression and note the presence of ECE forms with different molecular profiles [3, 4].

One of the main contributors to heterogeneity in malignant cells, even within the same morphological form of cancer, and ambiguous response to anticancer therapy is genome instability [5]. Replication stress (RS), characterized by disruptions of DNA structure during replication, is one of the key drivers of genome instability [6]. Structural abnormalities that can cause RS include ribonucleotide insertion instead of dioxyribonucleotides in the DNA chain, single- and double-strand breaks, presence of hairpins and overexpression of oncogenes, such as *KRAS*, *MYC*, and *cyclin E* [7]. Increased expression of the *KRAS* oncogene is identified in 22–43% of endometrial carcinomas [8] and leads to changes in the expression of *ATR* and *CHEK1* genes, key components of the ATR-CHEK1-WEE1 signaling pathway responsible for DNA replication and repair. In malignant cells, the ATM-CHEK2-p53-p21 pathway becomes inactivated [9]. In the absence of proper DNA repair or replication checkpoint response, genetic instability increases in tumor cells, contributing to uncontrolled proliferation and subsequent emergence of biologically polymorphic clones of tumor cells with increased invasive and metastatic potential, and resistance to therapy [10–12].

Therefore, assessing the RS value as an inducer of genomic damage and identifying biomarkers associated with DNA replication and repair will help clarify the phenomenological causality of these changes in the progression of endometrial tumors and may aid in finding molecular markers of cancer progression, predicting clinical outcome and identifying new therapy targets.

The aim of the current study is to evaluate the expression profile of *KRAS*, *ATR* and *CHEK1* genes at both mRNA and protein levels, which are associated with replicative stress in the progression of ECE in patients with stage I tumors.

## Materials and methods

### Samples

Archived samples, paraffin-embedded operative material from 40 patients with endometrial cancer (PE) stage I according to FIGO, aged 38 to 72 years (average 60.4±2.5 years), were used in this study. All patients did not receive preoperative therapy and were treated in the National Cancer Institute of the Ministry of Health of Ukraine in the period from January 15, 2014 to December 23, 2019 and provided informed written consent for the use of their tissue samples and clinical data for scientific purposes. The patients consent is recorded by the doctor in the medical history and certified by the patient’s personal signature. The archived samples were accessed for research purposes on January 4, 2022. Archival samples were coded with serial numbers, and authors did not have access to information that could identify individual participants during or after data collection.

According to the conclusion of the Bioethics Commission of the R.E. Kavetsky Institute of Experimental Pathology, Oncology and Radiobiology of the National Academy of Sciences of Ukraine (protocol No. 5 dated December 15, 2021), all necessary ethical standards were observed during the study in accordance with the requirements of universally recognized international rules within the framework of the Helsinki Declaration of 2008.

There are 20 samples of ECE obtained from patients without metastasis in regional lymph nodes during the first 3 years after primary treatment and 20 samples of ECE obtained from patients with metastasis in regional lymph nodes developed during the first 3 years after primary treatment (recurrence). The detailed characteristics of metastasis localization with indication of occurrence time indicated in Table 2 in S1 File. Morphological verification of the pathological process in the endometrium and the degree of tumor differentiation was performed on preparations stained with hematoxylin and eosin according to WHO recommendations [13].

### Immunohistochemical study

Immunohistochemical (IHC) detection of biomolecular marker expression was performed on deparaffinized sections of endometrial tumors. IHC was performed using primary antibodies against KRAS (Thermo Fisher Scientific, USA), phosphorylated (p) ATR (Ser428) (Thermo Fisher Scientific, USA), phosphorylated (p) CHEK1 (Ser296) (Thermo Fisher Scientific, USA). Visualization of these markers was performed using the PolyVue detection system (Diagnostic BioSystems, USA). The results of the immunohistochemical reaction were assessed using a semiquantitative method by counting the number of positively stained cells determined as a percentage—labeling index (LI, %). In each case, 800–1000 tumor cells were analyzed. LI values lower than median (Me) indicated low expression of the corresponding marker, while LI and PI values higher than Me indicated high expression.

### qRT-PCR

Real-time polymerase chain reaction (PCR) was used to assess mRNA expression levels. Total RNA was isolated from ECE samples using the commercial kit "For RNA isolation" (Ukrainian Genetic Technologies, Ukraine) according to the manufacturer’s protocol. The tissue samples were thoroughly crushed and incubated with 50 μl of protease K at +75°C for 15–30 minutes. Afterwards, 300 μl of lysis buffer was added followed by sample vortexing and incubation at +75°C for 15–30 min. Then, 400 μl of precipitation buffer was added followed by sample vortexing, and the resulting suspension was transferred to columns. The columns were washed twice, and RNA was eluted in 50 μl of sterile, DNAse- and RNAse-free water. Concentrations of the obtained RNA samples were measured on the spectrophotometer "ND 1000" using "NanoDrop 1000 v. 3.7" software (Thermo Scientific, USA) by measuring the absorbance ratio at wavelengths of 260 and 280 nm. Sample aliquots were made and stored at -70°C.

Two micrograms of isolated RNA were reverse transcribed to cDNA using RevertAid Reverse Transcriptase, RiboLock RNase Inhibitor, and Oligo(dT)18 anchored primer (all from Thermo Scientific, USA). qRT-PCR was performed using Maxima SYBR Green/ROX qPCR Master Mix (Thermo Scientific, USA) on the AppliedBiosystems 7900HT FastReal Time PCR System (Applied Biosystems, USA). The primer sequences used in this study are listed in Table 1. The following amplification conditions were used for all studied genes: (95°C, 10 min., 95°C, 15 sec., 62°C, 40 sec.) X (40 cycles) followed by a dissociation curve to determine the specificity of the amplified product (95°C, 15 sec., 64°C, 40 sec., 95°C, 15 sec.). Expression of *GAPDH* was used as an endogenous control for standardization. Ct values were determined for the internal control (GAPDH) and the test genes at the same threshold level in the exponential phase of the PCR curves. Relative quantification (comparative Ct (ddCt) method) was used to compare the expression level of the tested genes with an internal control and was represented in relative units. Dissociation curve analysis was performed after each run to check the specificity of the reaction. Three reactions (each in triplicate) were run for each gene, and the standard error of the mean was calculated.

**Table 1:** The primer sequences used in the study.

Gene target	Forward primers 5’→3’	Reverse primers 5’→3’
*GAPDH*	gtggacctgacctgccgtct	ggaggagtgggtgtcgctgt
*KRAS*	attccttttattgaaacatcagca	tcggatctccctcaccaat
*ATR*	tgcagtaatgtcaatggttgg	ctggaacttcaaaggtttctcc
*CHЕK1*	cggtggagtcatggcagtgccc	tctggacagtctacggcacgcttca

### Statistics

Statistical analysis was conducted using Statistica 7.0 software (StatSoft, Inc.), MedCalc® Statistical Software version 22.016 (MedCalc Software Ltd.) and GraphPad Prism8 (GraphPad Software) employing standard descriptive, non-parametric (Mann-Whitney test) and Spearman’s rank correlation coefficient methods. Differences at p<0.05 were considered significant. Receiver operating characteristic (ROC) curve analyses including calculations of sensitivities and specificities were used to investigate the prognostic ability of the studied biomarkers.

## Results

### *KRAS*, *ATR* and *CHEK1* mRNA expression levels in endometrial carcinomas of the endometrium

All analyzed ECE samples were from patients in stage I of tumor process and were categorized into two main groups based on recurrences detection: samples from patients with EC recurrence (metastases in regional lymph nodes detected within 1.8–36.0 months after the initial surgical treatment) and samples from patients without detected metastasis in regional lymph node named EC w/o recurrence.

Among patients without EC recurrence, G1-G2 tumors were identified in 70% of cases and invasion of the myometrium was found to be not deep (<1/2) in 75% of cases. Among patients with recurrent EC, poorly differentiated (G3) tumors were identified in 50% of cases, and 55.0% demonstrated deep (>1/2) invasion in the myometrium (Table 2).

**Table 2:** Clinicopathological characteristics of patients with ECE.

Indicator	Number of patients, N = 40	
	w/o recurrence, N = 20	recurrence, N = 20
Average age, years	60.4±2.5	
(min-max)	(38–72)	
Degree of differentiation,		
G1-2, N (%)	14 (70)	10 (50)
G3, N (%)	6 (30)	10 (50)
The depth of tumor invasion in the myometrium		
<1/2, N (%)	15 (75)	9 (45)
>1/2, N (%)	5 (5)	11 (55)

**Fig 1:**
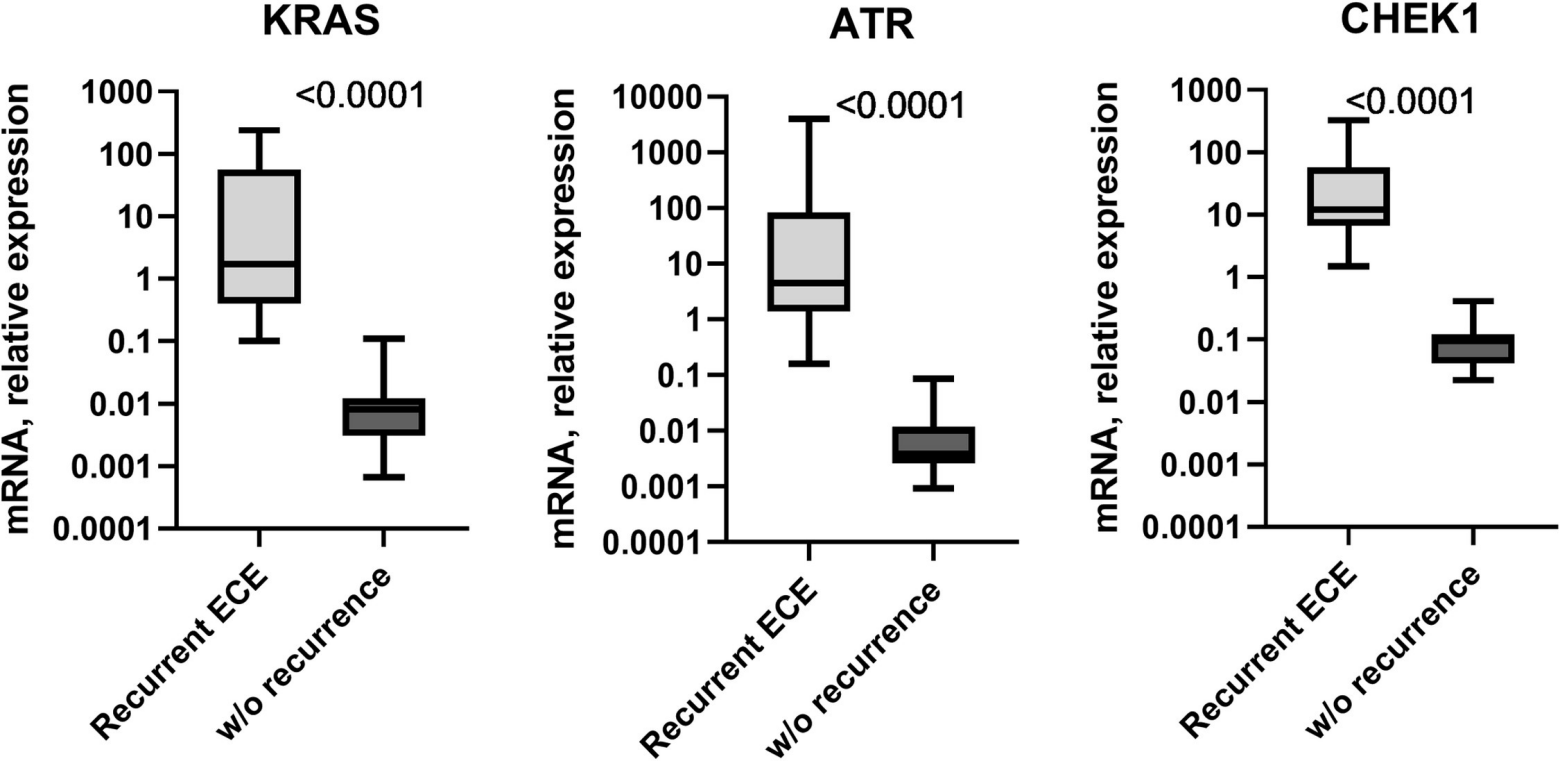
The mRNA expression levels of *KRAS*, *ATR* and *CHEK1* in endometrial cancer tissue depending on recurrence in anamnesis. Results of qRT-PCR. Expression levels of studies genes were normalized to *GAPDH* expression level. Box plots show quartiles, median, minimum and maximum values.

The mRNA expression profile of *KRAS*, *ATR* and *CHEK1* genes varied in ECE samples depending on the history of recurrence. The mRNA expression pattern of the studied genes was heterogeneous in recurrent ECE samples, while in samples without metastatic lesions expression levels were more uniform. In recurrent ECE samples, the median value of *KRAS* mRNA expression levels was 1.7 with a minimum and maximum value of 0.1 and 239.1, respectively. The *ATR* mRNA level was slightly increased compared to the *KRAS* mRNA expression level in recurrent ECE samples (Me = 4.5, minimum– 0.16, maximum– 4039). The *CHEK1* mRNA expression level with a median of 12.0 (minimum—1.5, maximum—326.4) was the highest among the studied genes. In ECE samples without relapse in patient’s anamnesis, the highest level of mRNA expression was specific to the *CHEK1* gene (median value = 0.1, minimum = 0.02, maximum = 0.4), which was significantly different from the level of *KRAS* (median value = 0.01, minimum = 0, maximum = 0.1) and *ATR* mRNA (median value = 0.01, minimum = 0, maximum = 0.08). The mRNA expression levels of *KRAS*, *ATR*, *CHEK1* genes were significantly higher in the samples of patients with recurrent ECE compared to those in the group of patients without recurrence (p<0.0001) (Fig 1). Thus, the mRNA levels of *KRAS*, *ATR*, *CHEK1* genes varied significantly in ECE of patients withrecurrence, while in the ECE group of patients without recurrence, the studied genes’ mRNA expression levels were more homogeneous. The *CHEK1* mRNA expression level was elevated in both groups of endometrial tumors compared to *KRAS* and *ATR* mRNA expression levels. Elevated levels of all three studied genes were specific features of recurrent ECE cases.

To elucidate the differences in *KRAS*, *ATR*, *CHEK1* mRNA expression levels between the two analyzed group of samples, we checked for their correlation with tumor grade differentiation and depth of myometrium invasion, which are key clinicopathological indicators of tumor progression. It was observed that the median values of *KRAS*, *ATR* and *CHEK1* mRNA expression levels in recurrent ECE were higher in samples with high (G1) and moderate (G2) differentiated grades compared to the expression levels in poorly (G3) differentiated ECs. However, the detected difference was statistically significant only for *ATR* (Table 3). There were no differences in *KRAS* and *ATR* mRNA expression levels based on the degree of tumor differentiation in EC samples without recurrence (Table 3). Conversely, in the ECE group without recurrence, significantly increased *CHEK1* mRNA expression levels were found in poorly differentiated tumors (G3) (Table 3). The mRNA expression levels of *KRAS*, *ATR*, *CHEK1* genes in EC tissue samples from both groups with and without recurrence were independent of the depth of tumor invasion into the myometrium (Table 3).

**Table 3:** The mRNA expression levels of *KRAS*, *ATR*, *CHEK1* genes in endometrial cancer tissue samples at stage I with and without recurrence in anamnesis.

Gene	Values	**Recurrent EC**			
		Differentiation grade		Depth of invasion	
		G1-G2	G3	<1/2	>1/2
*KRAS*	Median	28.83	1.340	8.15	1.34
	Min-max	0.4–112.6	0.1–21.50	0.1–112.6	0.16–56.25
*ATR*	Median	66.8	1.55*	7.1	2.1
	Min-max	1.8–656.3	0.16–82.90	0.2–656.3	0.3–128.8
*CHEK1*	Median	18.42	9.27	5.75	13.02
	Min-max	1.5–311.0	3.7–57.20	1.5–132,1	6.7–311.0
Gene	Values	**EC w/o recurrence**			
		Differentiation grade		Depth of invasion	
		G1-G2	G3	<1/2	>1/2
*KRAS*	Median	0.01	0.01	0.01	0,01
	Min-max	0–0,03	0–0,1	0–0,1	0–0,01
*ATR*	Median	0,004	0,004	0,004	0,01
	Min-max	0–0,08	0–0,04	0–0,1	0,002–0,02
*CHEK1*	Median	0,06	0,1*	0,1	0,1
	Min-max	0,02–0,2	0,1–0,4	0,02–0,4	0,03–0,17

Notes. *- p<0,05 compare to G1-G2 differentiation grade.

### The KRAS protein levels and the levels of phosphorylated forms of ATR and CHEK1 in endometrial carcinomas of the endometrium

**Fig 2:**
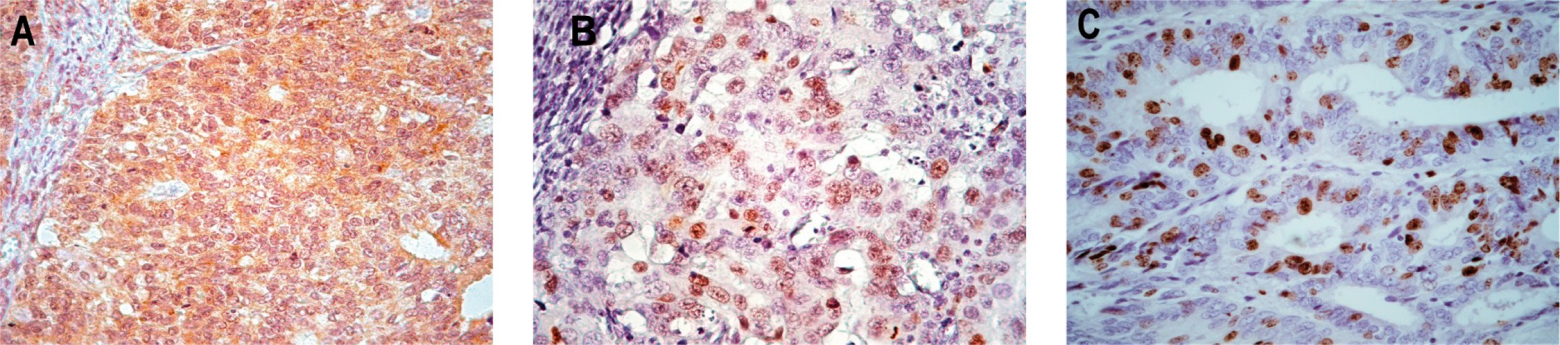
Representative microphotographs of KRAS (a), pATR (b) and pCHEK1(c) protein detection. (A), (B)—low differentiated ECE (× 400); (C)—moderately differentiated ECE (× 400). Immunohistochemical staining, additional staining by Mayer’s hematoxylin.

Next, we analyzed whether the differences detected in *KRAS*, *ATR*, *CHEK1* mRNA expression levels in ECE samples were also reflected at the protein levels. The IHC study established that the majority (94.8%) of EC showed positive expression of the KRAS oncoprotein. All studied ECEs were positive for the phosphorylated forms of ATR (pATR) and CHEK1 (pCHEK1) markers (Fig 2). At the same time, individual values of KRAS, pATR and pCHEK1 protein levels in endometrial carcinomas varied significantly ranging from 0 to 78.9%, 1.8 to 58.6% and 3.6 to 78.3%, respectively. The median expression levels of KRAS, pATR and pCHEK1 were 18.6%, 11.3% and 20.1%, respectively. At the same time, high (>Median value) expression levels of KRAS and pATR were observed in 50.0% of cases and of pCHEK1 in 53.0% of cases.

**Fig 3:**
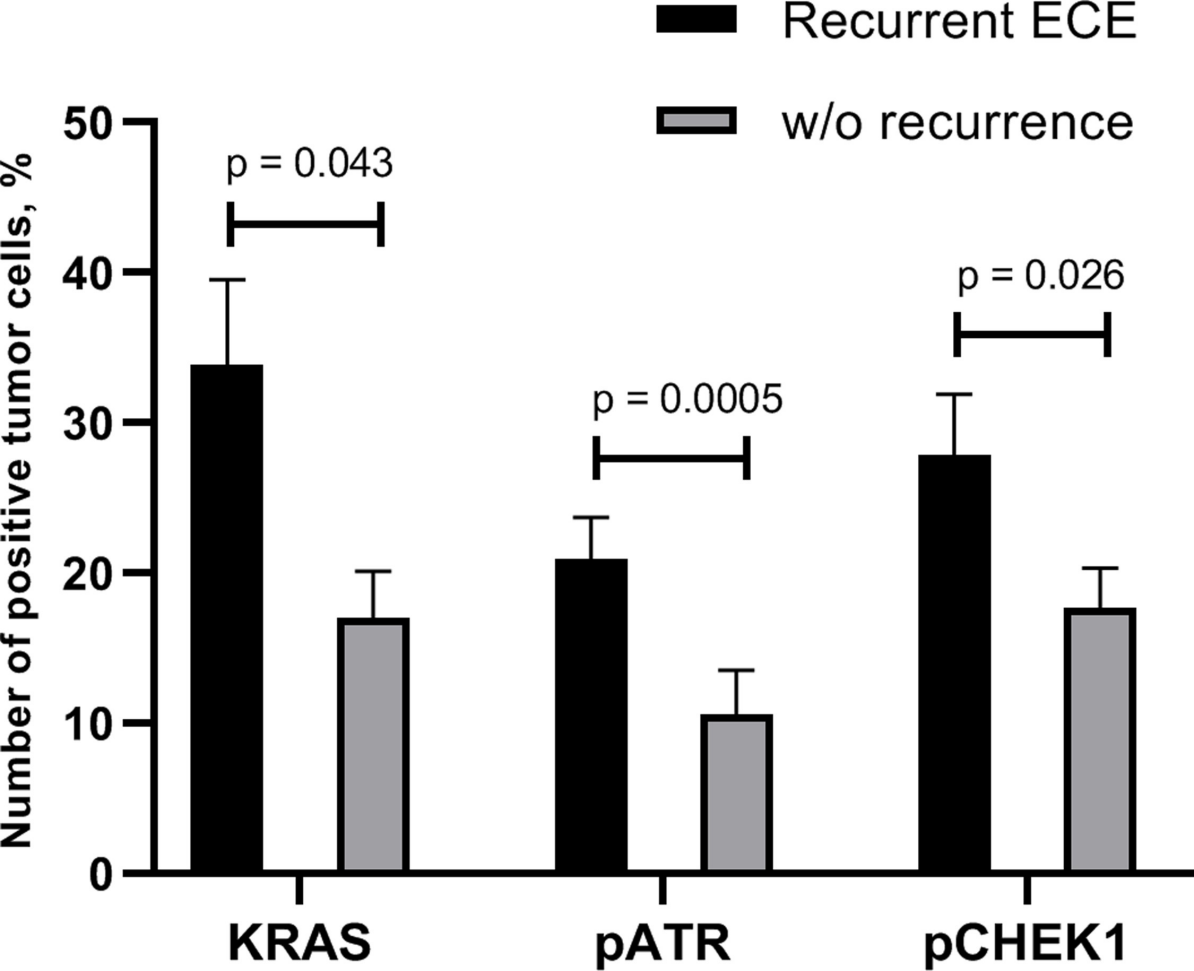
Amounts of KRAS, pATR and pCHEK1 positive tumor cells in ECE depending on recurrence incidences. Semiquantitative analysis of immunohistochemical staining results by counting the number of positively stained cells presented as labeling index (LI, %). Graphs showed Mean±SEM.

Significantly higher protein levels of KRAS, pATR and pCHEK1 were detected in the group of ECE with recurrences (2.0, 2.0 and 1.6 times higher, respectively) compared to ECE cases without recurrences (Fig 3). The levels of the KRAS oncoprotein were higher in both groups of endometrial carcinomas compared to the level of pATR and pCHEK1 proteins. High levels (>Me value) of all three studied proteins were a distinguishing feature of recurrent ECE. A reliable direct correlation between the level of mRNA and protein expression of KRAS, pATR, pCHEK1 was found in the ECE of patients both without and with recurrences (R = 0.6; 0.9; 0.9 and R = 0.9; 0.7; 0.7 respectively).

In the group of recurrent ECE, the median value of pATR expression levels was significantly reduced in tumor samples with a G3 differentiation grade compared to G1-2, while a high pCHEK1 protein level was specific to ECE cases with deep tumor invasion in the myometrium (>1/2) (Table 4). Statistically higher (2.9-fold) levels of KRAS, and elevated expression of pATR (2.8 fold) and pCHEK1 (1.3-fold) proteins were detected in poorly differentiated endometrial carcinomas in the group without recurrences compared to neoplasms with high and moderate grades of differentiation (Table 4). In endometrial tumors that deeply invaded the myometrium, a 3-fold increase in the KRAS protein level was observed, along with a slightly higher pATR expression (2.1-fold), while the number of pCHEK1 positive cells did not depend on the depth of tumor invasion into the myometrium.

**Table 4:** The biomarkers expression at the protein level in ECE tissue samples categorized by recurrence incidences and tumor clinicopathological characteristics.

Protein	Values (%)	**Recurrent EC**			
		Differentiation grade		Depth of invasion	
		G1-G2	G3	<1/2	>1/2
KRAS	Median	35.9	18.5	20.1	21.6
	Min-max	10.3–78.9	3.6–62.3	3.6–72.7	5.4–78.9
pATR	Median	25.5	14.0^*^	18.2	16.8
	Min-max	8.4–58.5	5.6–29.8	5.6–36.0	10.2–58.5
pCHEK1	Median	27.3	24.2	13.9	26.3^#^
	Min-max	5.4–78.3	8.2–49.8	5.4–41.2	19.4–78.3
Protein	Values (%)	**EC w/o recurence**			
		Differentiation grade		Depth of invasion	
		G1-G2	G3	<1/2	>1/2
KRAS	Median	7.6	22.0^*^	7.8	23.5^*^
	Min-max	0–42.3	18.6–51.4	0–51.4	18.8–30.9
pATR	Median	4.2	12.1	4.5	9.3
	Min-max	1.8–58.6	3.7–22.6	1.8–58.6	3.7–18.6
pCHEK1	Median	14.5	18.7	18.5	15.8
	Min-max	3.6–32.3	13.2–56.2	3.6–56.2	13.2–19.6

Notes. * p < 0.05 compared to G1-G2 differentiation grade

# p < 0.05 compared to <1/2 depth of invasion.

**Fig 4:**
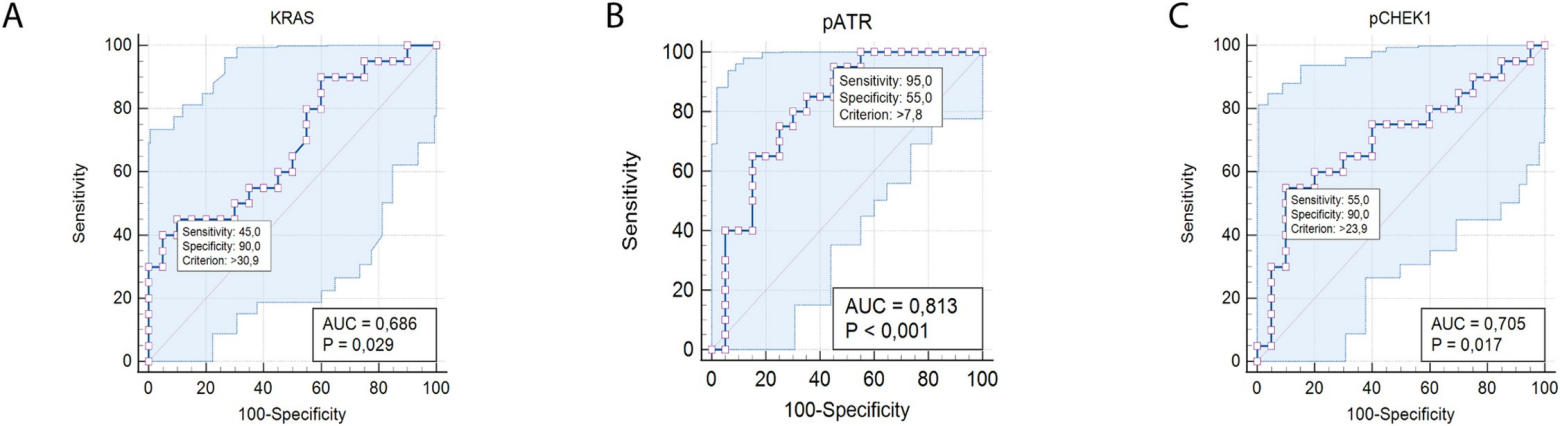
The ROC analysis of KRAS, pATR and pCHEK1 prognostic biomarkers. The ROC curves demonstrate the sensitivity and specificity of KRAS (A), pATR (B) and pCHEK (C) for predicting recurrence in ECE at stage I.

To evaluate the prognostic potential of KRAS, pATR and pCHEK in ECE at stage I, the AUC (area under the receiver operating characteristic curve) values of the receiver operating characteristic (ROC) curves were calculated (Fig 4). The AUC values for KRAS, pATR, and pCHEK were 0.686, 0.813, and 0.705, respectively. The highest AUC value was observed for pATR, indicating a sensitivity of 95.0% and a specificity of 55.0% in discriminating between cases with and without metastases (Fig 4B).

Therefore, distinct differences in the molecular profile of ECE at the first stage of tumor process have been established. ECE cases with recurrence are characterized by poorly differentiated tumors that deeply invade the myometrium, which correlates with increased expression of the oncoprotein KRAS and markers associated with replicative stress—pATR and pCHEK1. Heterogeneity in the protein levels of KRAS, pATR and pCHEK1 was observed both in the ECE group of patients without recurrence and those with recurrence. Elevated levels of KRAS protein are characteristic of poorly differentiated grade tumors with deep myometrium invasion of endometrial carcinomas in the group of patients without recurrence in anamnesis. Whereas in the group of patients with recurrence, higher levels of KRAS, pATR and pCHEK1 were observed in G1-G2 endometrial carcinomas with statistically significant differences noted for pATR. High pCHEK1 protein levels are associated with deep tumor invasion into the myometrium in the group of patients with recurrence. ROC analysis confirmed the specificity and sensitivity of KRAS, pATR and pCHEK1 evaluation as predictors of recurrence development in patients with ECE, with the pATR marker demonstrating the highest prognostic value among others.

## Discussion

One of the hallmarks of cancer is genomic instability, which can be primary or arise from both exogenous and endogenous factors, leading to the activation of oncogenes and the inactivation of tumor-suppressor genes [14]. At the same time, the so-called "replicative stress of DNA" plays a key role in generating genomic changes. Deficiencies in the functioning of replicative stress response proteins can lead to the accumulation of DNA errors, loss of genomic integrity, and result in either cell death or malignant transformation and progression [10]. The ATR-CHEK1 signaling pathway plays a critical role in responding to replicative stress [15] through the initiation of DNA repair mechanisms. Specifically, the signaling pathway strongly inhibits cyclin-dependent kinase activity and cell cycle progression, induce the p53 transcription factor, recruits repair factors to damaged DNA, and activates DNA-repair proteins via post-translational modifications including phosphorylation, acetylation, ubiquitination, or sumoylation. In cancer, upregulated oncoproteins KRAS, MYC, CCNE1, etc. act as primary sources of replicative stress, while also impairing the cell’s ability to adequately respond to such stress.

In our study, we observed upregulated expression of the KRAS oncogene and markers associated with replicative stress, ATR and CHEK1, at both mRNA and protein levels in the group of ECE samples from patients with recurrence. Moreover, increased levels of KRAS, ATR, and CHEK proteins correlated with a high rate of tumor cell proliferation and aneuploidy in ECE cases with metastatic lesions following primary tumor resection. Our previous study showed that the studied groups of ECE cases are significantly heterogeneous in terms of cells ploidy. All examined tumor cells (100%) in ECE samples obtained from patients without metastasis in anamnesis were diploid, while in recurrent ECE cases both diploid (80%) and aneuploid (20%) neoplasms were identified. Significantly elevated levels of KRAS, pATR and pCHEK1 proteins were found in endometrial carcinomas with aneuploidy compared to diploid tumors (p < 0.05) [16]. Therefore, the detected changes in the expression of biomolecular markers associated with replicative stress correlate with the proliferative potential of tumor cells, aneuploidy and the appearance of metastasis in patients with ECE.

It is important to note that in analyzed ECE samples obtained from patients without recurrences, significantly elevated levels of KRAS protein and CHEK1 mRNA were observed in poorly differentiated (G3) tumors. In ECE cases with recurrences, poorly differentiated (G3) endometrial carcinomas were characterized by downregulated mRNA levels of *ATR* and pATR protein. Deeply invaded recurrent ECE could be distinguished based on increased CHEK1 mRNA levels and their phosphorylated protein form.

Evaluating the replicative stress response including key signaling components in tumors of different tissue origins is important for two main reasons: prognostic/predictive value and targets for the precision therapy. ATR and CHEK1 expression levels are often elevated in many forms of cancer, including ovarian, breast and prostate cancer and correlate with poor outcomes for patients [17, 18]. A clinical study has shown that low cytoplasmic levels of pATR are associated with an advanced stage, serous histology, and high preoperative serum CA125 concentrations in patients with epithelial ovarian cancer, which is also linked to poor disease-free and overall survival [19]. In breast cancer, high ATR expression levels were associated with high tumor stage, high tumor grade, a high mitotic index, polymorphisms, and lymphovascular invasion [20]. In our study, the analyzed pATR was predominantly found in the cell nucleus of ECE. The decreasing mRNA expression and pATR protein levels in poorly differentiated tumors of recurrent ECE cases may indicate a full disruption of the ATR-CHEK1 defense system with tumor progression. The presence of mutations in the ATR gene in combination with oncogenic stress caused by high expression of KRAS can cause synthetic lethality of tumor cells or contribute to the loss of functioning of tumor suppressor genes, particularly TP53, and increased genomic instability [21, 22]. High expression of the ATR gene and its protein product may result from the influence of dominant negative mutations, which function as dominant negative inhibitors of ATR function leading to the accumulation of an inactive form of the indicated protein [23]. Typically, when ATR is inhibited, there is also a decrease in CHEK1 activity. However, CHEK1 can be activated by other kinases. In addition to ATR, CHEK1 activation can also be provided by EZH2. EZH2 was found to be important in promoting resistance to chemotherapy in ovarian cancer cells, as well as their ability to actively proliferate [24, 25]. The mRNA and protein levels of ATR and CHEK1 identified in recurrent ECE samples may also indicate the involvement of additional regulatory pathways possibly including specific miRNA. Elevated mRNA and protein levels of CHEK1 in deeply invaded recurrent ECE may indicate CHEK1’s dominant role in ECE progression, while in ECE cases without metastasis ATR and CHEK1 expression levels were similar.

Association between KRAS, ATR and CHEK1 expression and overall survival of EC patients are often conduct in line of determine the KRAS, ATR and CHEK1 mutation, but nor general mRNA or protein expression. Thus, ATR mutation are the most common in EC compared to the other cancer type [26]. The study with involvement of 475 EC patients showed that ATR mutation was specific for microsatellite instability EC subgroup and are associated with high-grade endometrial cancers, without confirmed correlation with overall survival [27]. In contrast, other study indicates that truncating ATR mutations in EC are associated with reduced disease-free and overall survival [28]. Interestingly that KRAS amplification but not mutation was associated with aggressive phenotype [29]. High CHEK1 expression link to decreased risk of progression and death, after adjusting for stage [30]. During DNA replication, tumor cells activate a number of mechanisms that contribute to DNA restoration, proliferation and apoptosis tolerance. Therefore, the development of approaches directed to the inhibition of ATR and CHEK1 in malignant neoplasms, particularly in ECE, could increase the effectiveness of therapeutic measures to prevent recurrence/metastases from occurring.

## Conclusion

For the first time, an association between components of the KRAS/ATR/CHEK1-signaling pathway, responsible for coordinating replicative stress, and markers of tumor progression, including invasiveness and tumor recurrence, has been established in patients with stage I ECE. This finding indicates the pathogenic role of aberration in DNA replication during tumor progression and enables the identification of groups of patients with a high possibility of tumor relapse accompanied by the metastatic process. The obtained data can be used as a foundation for identifying more aggressive forms of ECE and will serve as a basis not only for early diagnosis and prognosis but also help improve therapy effectiveness through personalized treatment strategies.

## Supporting information

S1 File The minimal data set. (XLSX)
